# Structural determination of bilayer graphene on SiC(0001) using synchrotron radiation photoelectron diffraction

**DOI:** 10.1038/s41598-018-28402-0

**Published:** 2018-07-05

**Authors:** I. Razado-Colambo, J. Avila, D. Vignaud, S. Godey, X. Wallart, D. P. Woodruff, M. C. Asensio

**Affiliations:** 1 Synchrotron SOLEIL & Université Paris-Saclay, 91192 L’Orme des Merisiers, Saint Aubin-BP 48, Gif sur Yvette Cedex, France; 2 Institute of Mathematical Sciences and Physics, University of the Philippines Los Baños, Laguna, 4031 Philippines; 3 Institut d’Electronique, de Microélectronique et de Nanotechnologie (IEMN UMR 8520), Université Lille, CNRS, Centrale Lille, ISEN, Université Valenciennes, Villeneuve d’Ascq Cedex, 59652 France; 4 Physics Department, University of Warwick, Coventry, CV4 7AL United Kingdom

## Abstract

In recent years there has been growing interest in the electronic properties of ‘few layer’ graphene films. Twisted layers, different stacking and register with the substrate result in remarkable unconventional couplings. These distinctive electronic behaviours have been attributed to structural differences, even if only a few structural determinations are available. Here we report the results of a structural study of bilayer graphene on the Si-terminated SiC(0001) surface, investigated using synchrotron radiation-based photoelectron diffraction and complemented by angle-resolved photoemission mapping of the electronic valence bands. Photoelectron diffraction angular distributions of the graphene C 1s component have been measured at different kinetic energies and compared with the results of multiple scattering simulations for model structures. The results confirm that bilayer graphene on SiC(0001) has a layer spacing of 3.48 Å and an AB (Bernal) stacking, with a distance between the C buffer layer and the first graphene layer of 3.24 Å. Our work generalises the use of a versatile and precise diffraction method capable to shed light on the structure of low-dimensional materials.

## Introduction

Graphene has attracted considerable interest in recent years in the scientific, technological and industrial communities due to its novel physical and electronic properties, which offer the possibility of fabricating new devices for future carbon-based nanoelectronics^1^. Graphene comprises a single layer of sp^2^-bonded carbon atoms that are organised in an open hexagonal network. The electronic band structure of graphene shows linear dispersion around the Fermi energy at the K-point of the surface Brillouin zone, instead of the parabolic dispersion that is observed in graphite. However, its detailed electronic band structure depends on several factors such as the number of layers, the symmetry of the lattice, the stacking ordering and their interlayer distances^2–4^. Here we report the results of a determination of the structural parameters, namely the stacking order and interlayer distances, of bilayer graphene grown on the Si-terminated face of SiC(0001). While bulk graphite is known to have the AB stacking structure and an interlayer distance of 3.35 Å, these parameters have been the subject of some debate for bilayer and multilayer graphene.

In recent years, a number of different methods of graphene production have emerged, each method resulting in different structural and electronic properties. Conceptually, the simplest such method is mechanical exfoliation, pioneered by Novoselov and Geim^5^. Prepared in this way the material has the same AB stacking between layers as bulk graphite. Graphene can be also grown by chemical vapour deposition (CVD) onto a range of substrates such as Cu and Ni, the resulting structural properties being strongly dependent on the growth conditions. Epitaxial growth of graphene on SiC is an alternative approach capable of producing large scale, high quality, graphene films that cannot be obtained by mechanical exfoliation. Graphene grown on the two polar faces (Si-face and C-face) of SiC had been thought to have different stacking sequences that would result in different electronic properties. Low energy electron diffraction (LEED) patterns of graphene on the Si-terminated Si-face appear sharp, but LEED patterns obtained from graphene on the C-face are generally smeared out into a continuous diffraction ring (albeit with some evidence of preferred rotation angles), implying contributions from either twisted adjacent layers or adjacent AB-stacked grains of different azimuthal directions. The electronic valence band structure of bilayer graphene on the Si-face shows splitting into two π band branches, but bilayer graphene on the C-face was commonly found to show linear dispersion near the Dirac point, as in monolayer graphene. However, angle-resolved photoelectron spectroscopy (ARPES) from single μm-sized grains has recently shown that multilayer graphene on the C-face has the same stacking and electronic structure as on the Si-face and also shown how different stacking sequences strongly influence the electronic structure^6–8^. There have been different theoretical predictions regarding the structural model of stacked graphene namely; AB stacking, AA stacking and twisted graphene layers^9,10^. Here we have investigated the alternative AB and AA stacking models and show that only AB stacking of bilayer graphene on the Si-face is consistent with our experimental data. In AB stacking, the two adjacent layers are displaced laterally exactly as in bulk graphite, whereas in AA stacking the carbon atoms of two adjacent layers have exactly the same lateral positions

Another important structural parameter in graphene is the spacing between the graphene layers. Density functional theory (DFT) calculations by Guo *et al*.^11^ revealed the possibility of tuning the energy gap of bilayer graphene via its interlayer spacing. The calculation showed that a relatively small variation of the interlayer spacing can result in significant changes in the field-induced gap. It is therefore interesting to determine the interlayer distances between graphene layers in order to provide key information required to understand the electronic properties of multilayer graphene as well as of graphene heterostructures with other two-dimensional materials^12^. There have been a number of theoretical and experimental studies that have sought to establish the interlayer spacings of multilayer graphene. DFT calculations based on the generalized gradient approximation (GGA) indicated that the interlayer distance of AB stacked bilayer graphene is larger than that of bulk graphite, and was predicted to be 3.58 Å^13^. Another DFT study, which included the effect of van der Waals dispersion forces, obtained an interlayer distance of 3.49 Å for AB-stacked bilayer graphene^14^, while the value found in the calculations of Guo *et al*. was 3.34 Å^11^. The only experimental investigation sharing the same goals involved surface x-ray reflectivity by Hass *et al*.^15^ was directed particularly to understanding the structure of the SiC/graphene interface; the results included a determination of the interlayer distance between the carbon buffer layer (that lies between the SiC and the graphene) and the first graphene layer of 2.32 Å, but also the interlayer spacings of the first and second graphene layers (3.5 Å) and between successive graphene layers (3.35 Å). However, the signal from the graphene layers was particularly weak compared to the spots originated by the massive SiC substrate, naturally indissociable in experiment. Ferrah *et al*.^16^ reported the results of an X-ray photoelectron diffraction (XPD) of ‘few layer graphene’ (FLG) on SiC(0001) but their study focused on the epitaxial relationship between FLG and the SiC substrate and not on the interlayer spacings. Another photoelectron diffraction study by Lima *et al*.^17^ was described by the authors as XPD, but was actually a study of the angular distribution of photoelectrons at kinetic energies of only ~160 eV, comparable to the work we report here; the XPD name is normally reserved for experiments with much higher photoelectron energies when the angular distribution is dominated by zero-order forward ‘focussing’ diffraction, whereas at much lower energies both forward scattering and backscattering can be important. However, this work by Ferrah *et al*. was focused on the structure of the carbon buffer layer, although they also studied one preparation with monolayer graphene. Their results showed that the buffer layer is formed by two domains rotated by 60^◦^ with respect to each other, the distance between the buffer layer and the graphene layer being found to be 3.75 Å.

The objective of the investigation reported here was to determine the stacking sequence of bilayer graphene and the associated interlayer spacings in an effort to reconcile the different values found in existing theoretical and experimental literature. To achieve this goal we used angle-scan photoelectron diffraction (PED) to study single-domain graphene bilayers grown on the Si-terminated SiC(0001) surface. In this technique, the intensity of a core level photoemission peak is measured as a function of the emission angle at fixed values of the photoelectron kinetic energy. Coherent interference of the directly emitted photoelectron wavefield with other components elastically scattered by the surrounding atoms leads to an angular distribution measured outside the surface that is characteristic of the local atomic environment of the emitter atoms. This structure can be determined by comparison of the experimentally-measured angular distributions with the results of multiple scattering simulations for different possible trial structures. Trial and error iteration of the model structure is then undertaken to achieve the optimal agreement between theory and experiment.

## Results and Discussion

**Figure 1:**
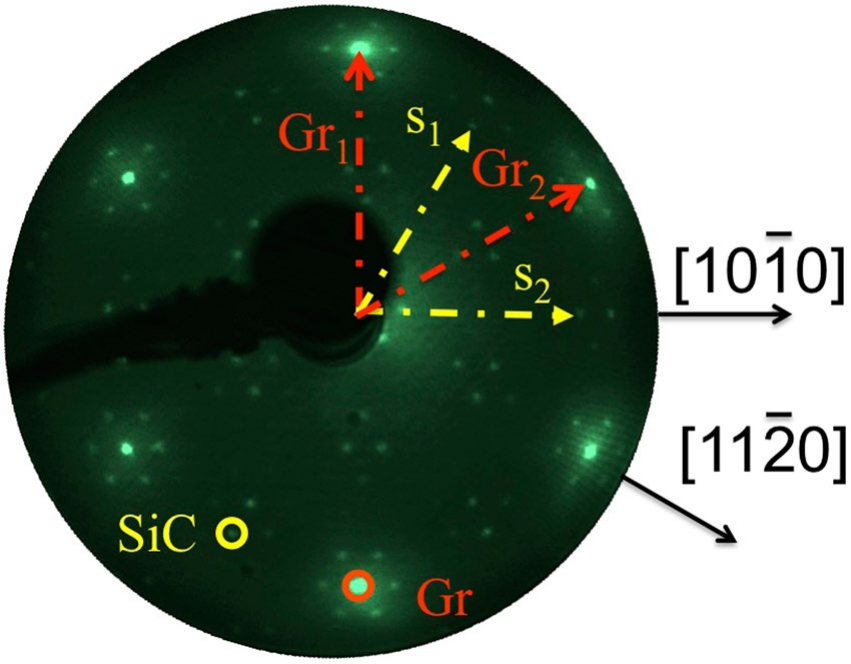
(6√3 × 6√3)R30° LEED pattern of the bilayer graphene on SiC(0001) at 91 eV electron energy. The primitive translation vectors of the reciprocal meshes of the SiC (S) and graphene (Gr) are superimposed while two associated first-order diffracted beams are circled.

**Figure 2:**
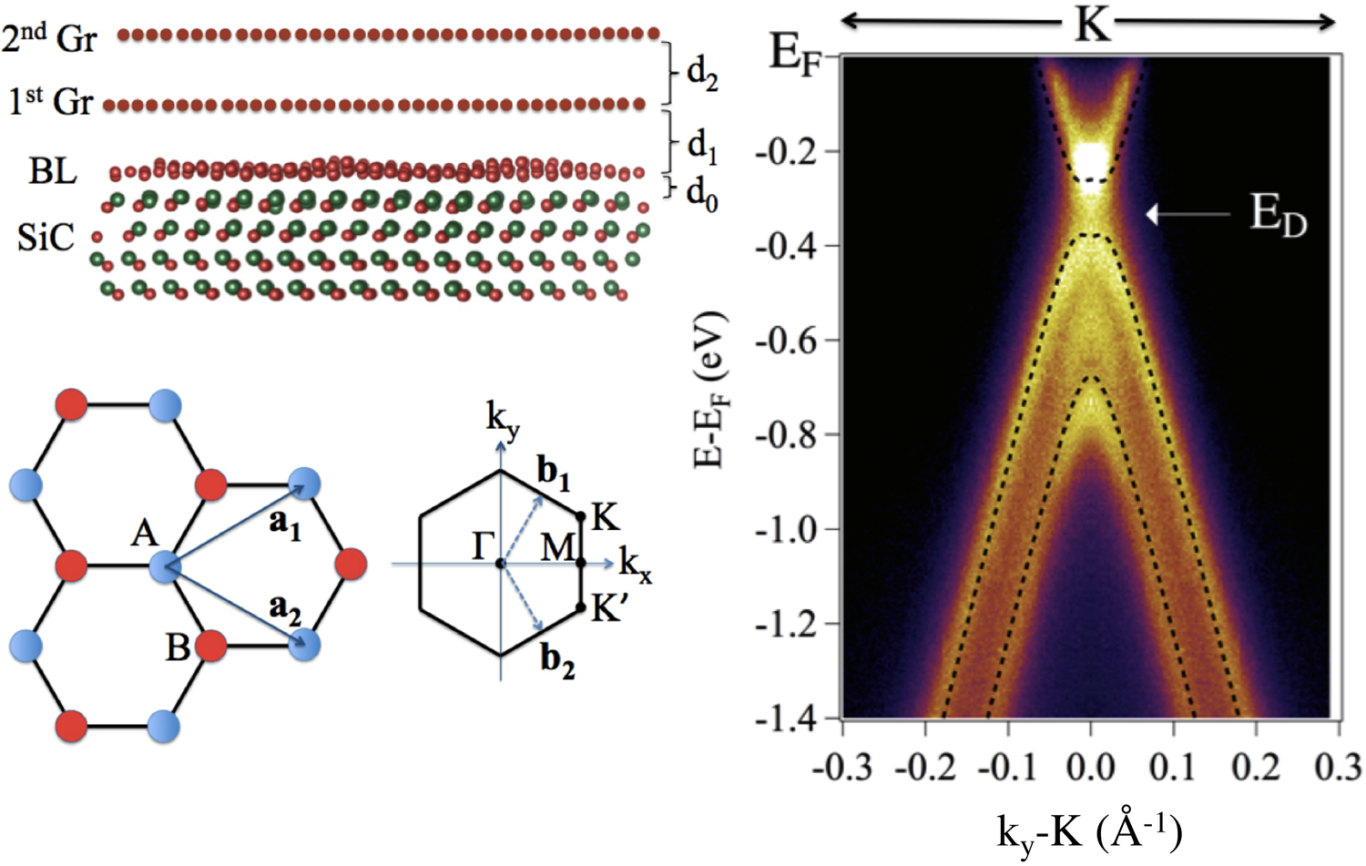
ARPES image of the valence band structure measured at the K point of the graphene surface Brillouin Zone along the direction perpendicular to the Γ-K line using 100 eV photon energy. The Dirac point is located 320 meV below E_F_.

Figure 1 shows the LEED pattern from the sample obtained after the graphitisation procedure described in the previous section. The diffraction pattern shows the (6√3 × 6√3)R30° reconstruction typically observed for monolayer and bilayer graphene grown on the Si-face of SiC(0001). The large unit cell of this reconstruction is required to achieve commensuration of the lattice parameters of graphene (2.46 Å) and the SiC substrate (3.08 Å), but is already created by the carbon ‘buffer layer’ that forms prior to the growth of the overlayer graphene. The (6√3 × 6√3)R30° mesh implies that the buffer layer and succeeding graphene layers are all rotated by 30° with respect to the SiC substrate. The buffer layer is well-established to serve as a precursor to the subsequent growth of graphene and comprises C atoms arranged in a honeycomb lattice (see Fig. 2). But unlike graphene, it is strongly bonded to the underlying SiC and does not possess the typical electronic properties of graphene, notably the linear band dispersion at the K point. This layer may be reversibly turned into graphene by hydrogen intercalation^18^.

The quality and character of the graphene deposition in our experiments, including the number of layers, is provided by an ARPES measurement of the valence band structure. The ARPES ‘image’ of the valence band structure of bilayer graphene measured perpendicular to the Γ-K direction at a photon energy of 100 eV is shown in Fig. 2. The two bilayer graphene π bands are shown in this figure, together with the fitted tight-binding calculated bands. Splitting of the π band originates from the interlayer interaction between the two graphene layers.

The Dirac point (E_D_) is located −320 meV relative to the Fermi level (E_F_) due to charge transfer from the substrate to the graphene layer that causes it to be n-doped. There is a 120 meV gap, as expected in the bilayer graphene on SiC(0001), due to the electric dipole that exists between the graphene and SiC interface; this induces an electrostatic asymmetry between the layers^4,19^. These electronic features displayed in Fig. 2 confirm that the graphene sample is a single domain bilayer.

**Figure 3:**
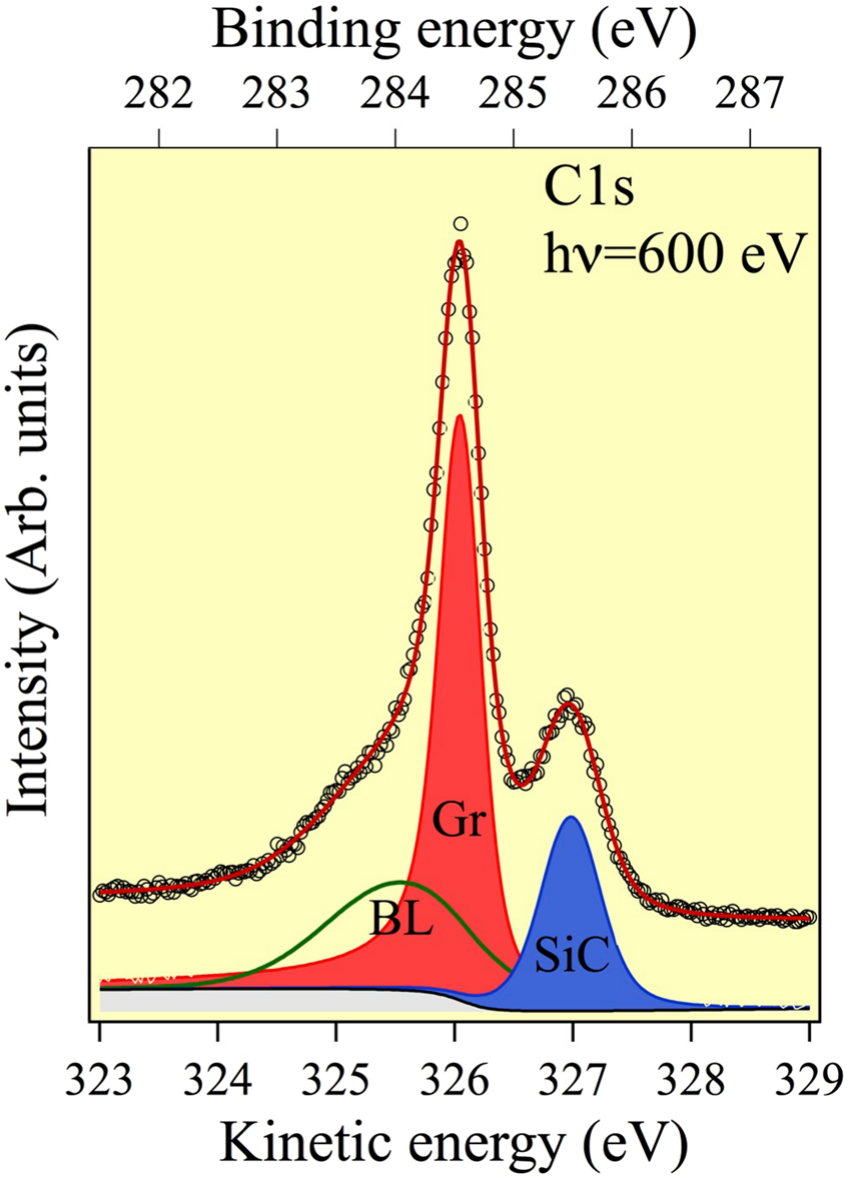
C 1s core-level photoemission spectrum obtained at photon energy of 600 eV. The best-fit curve and its chemically resolved components are superimposed.

Figure 3 shows a typical C 1s photoemission spectrum taken at a photon energy of 600 eV from the bilayer graphene. The spectrum is similar to that reported by Riedl *et al*.^18^. There are three components, labelled SiC, Gr, and BL. The SiC component arises from C atoms in the underlying bulk SiC, Gr is attributed to emission from the graphene layer, while BL is attributed to emission from the C in the interfacial boundary layer. The Gr component was fitted with a Doniach-Sunjic function to account for the metallic behaviour and the other components were fitted with a Voigt function. The Lorentzian widths were all set to 230 meV and the asymmetry parameter used for the graphene component was 0.06. The values for the Gaussian width are 267 meV (Gr), 460 meV (SiC), and 1.36 eV (BL). Photoelectron diffraction data were extracted from the intensity modulations of the graphene C 1s component (Gr) measured at different azimuthal and polar emission angles.

**Figure 4:**
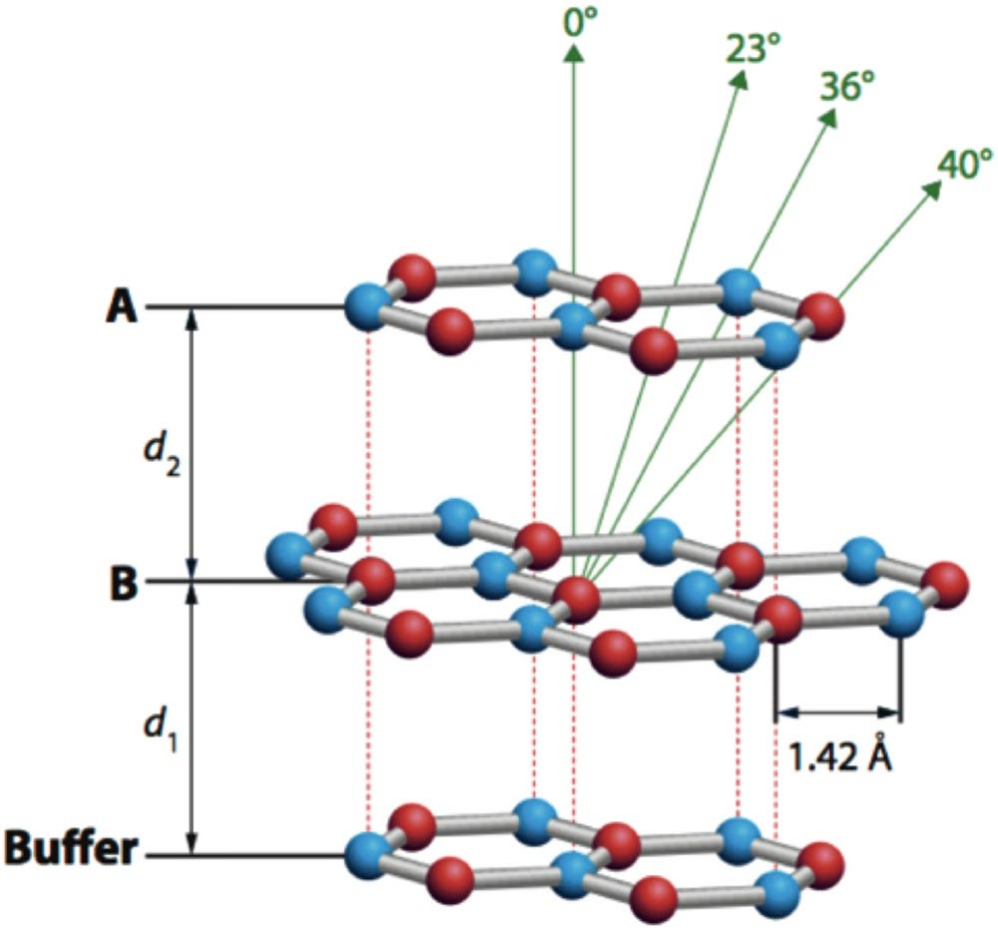
Atomic plane arrangement of bilayer graphene with AB stacking showing the different near-neighbour interlayer forward scattering directions. The angles shown correspond to the layer spacing of bulk graphite.

The atomic plane arrangement of bilayer graphene with AB stacking is depicted in Fig. 4. The labelled polar angles of the near-neighbour interlayer forward focusing directions correspond to the 3.354 Å interlayer spacing of bulk graphite, with in-plane C-C distances of 1.42 Å. The AB stacking structure consists of planes of C atoms each forming a hexagonal net stacked in a manner such that half of the C atoms are located directly above each other in adjacent planes, while the other half are located above the centres of the hexagon in the adjacent plane. The open hexagonal net structure leads to two inequivalent C atoms per unit cell, referred to as the α and β type atoms. The distance between the buffer layer and the first graphene layer is denoted as *d*_1_ and the distance between the first and second graphene layers is defined as *d*_2_. The AB stacking model is one of the two possible stacking sequences considered in the multiple scattering PED calculations, discussed in the succeeding sections.

The PED measurements of the graphene C 1s component presented here were obtained at two photon energies (hν), 600 and 900 eV, which correspond to 326.1 and 638.6 eV photoelectron kinetic energies, respectively. The photoelectron diffraction technique is typically performed in two different ranges of photoelectron kinetic energy that strongly influences the dominant scattering paths that contribute to the measured angular distribution. At high kinetic energies backscattering is weak and the dominant scattering processes are forward scattering zero-order diffraction with scattering angles close to zero; these ‘forward focussing’ peaks in the angular distribution correspond to interatomic directions. At low kinetic energies backscattering is much more significant (though forward scattering is still strong), allowing the technique to be used to locate adsorbate atoms relative to the underlying solid on which they are adsorbed. ‘High’ and ‘low’ typically means greater than, or less than, approximately 500 eV, although there is no sharp change in behaviour at this energy. The data we present here are thus recorded at intermediate energies in this range, but one may expect the higher-energy data to be more strongly influenced by forward scattering^20^. Summarizing, as at high kinetic energy, forward scattering is dominant, the evaluation of the photoelectron diffraction patterns of conveniently selected carbon emitters at the graphene bilayer provide precise structural information of the lattice bilayer symmetry. On the contrary, at low kinetic energy, since the backscattering is significant, the photodiffraction modulations are particularly sensible to rather small variations of the interlayer spacing of the graphene bilayer and the distance to the buffer layer.

**Figure 5:**
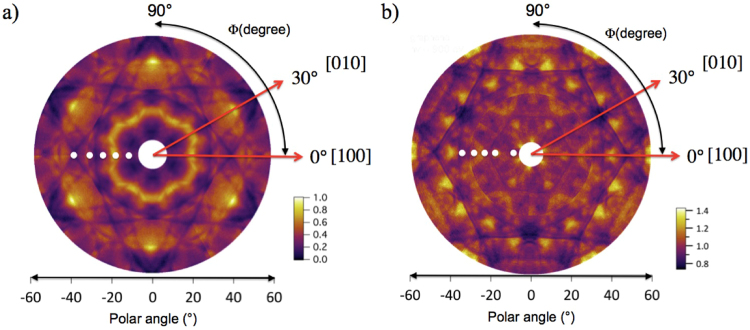
Experimental photoelectron diffraction angular distributions from the graphene C 1s component at photoelectron kinetic energies (KE) (**a**) 326.1 and (**b**) 638.6 eV. White spots are superimposed at polar emission angles of 12.2°, 20.2°, 26.7°, 32.7° and 37.5°, from the inner to the external part of the pattern, respectively.

The PED angular distributions of the graphene component of the C 1s photoemission recorded at the two different photon energies are shown in Fig. 5. These were constructed from data sets recorded in either π or 2π/3 azimuthal ranges, sufficient to confirm the presence of symmetrically equivalent regions. The PED distribution at the lower kinetic energy of 326.1 eV is shown in Fig. 5(a), while Fig. 5(b) shows the distribution at the photoelectron kinetic energy of 638.6 eV. Both clearly show the expected symmetry of a well-ordered graphene bilayer crystal; at first glance this appears to be 6-fold symmetric, but more careful inspection, possible in the extracted azimuthal plots shown below, reveals the true symmetry to be 3-fold with a mirror plane (3 m symmetry). The character of the distributions is rather different, with the lower energy distribution showing bright streaks whereas the higher energy pattern has more clearly-defined narrow peaks. The higher energy distribution does not show very bright sharp peaks common in many XPD experiments and associated with interatomic directions, but this can be partly accounted for by the relatively low energy but also the weak scattering of the low atomic number C atoms. Indeed, even at higher kinetic energy (964 eV) C 1s XPD from graphite does not display very strong forward scattering peaks^21^. The lower energy distribution is expected to be more strongly influenced by backscattering that can prove to be sensitive to the detailed structure.

**Figure 6:**
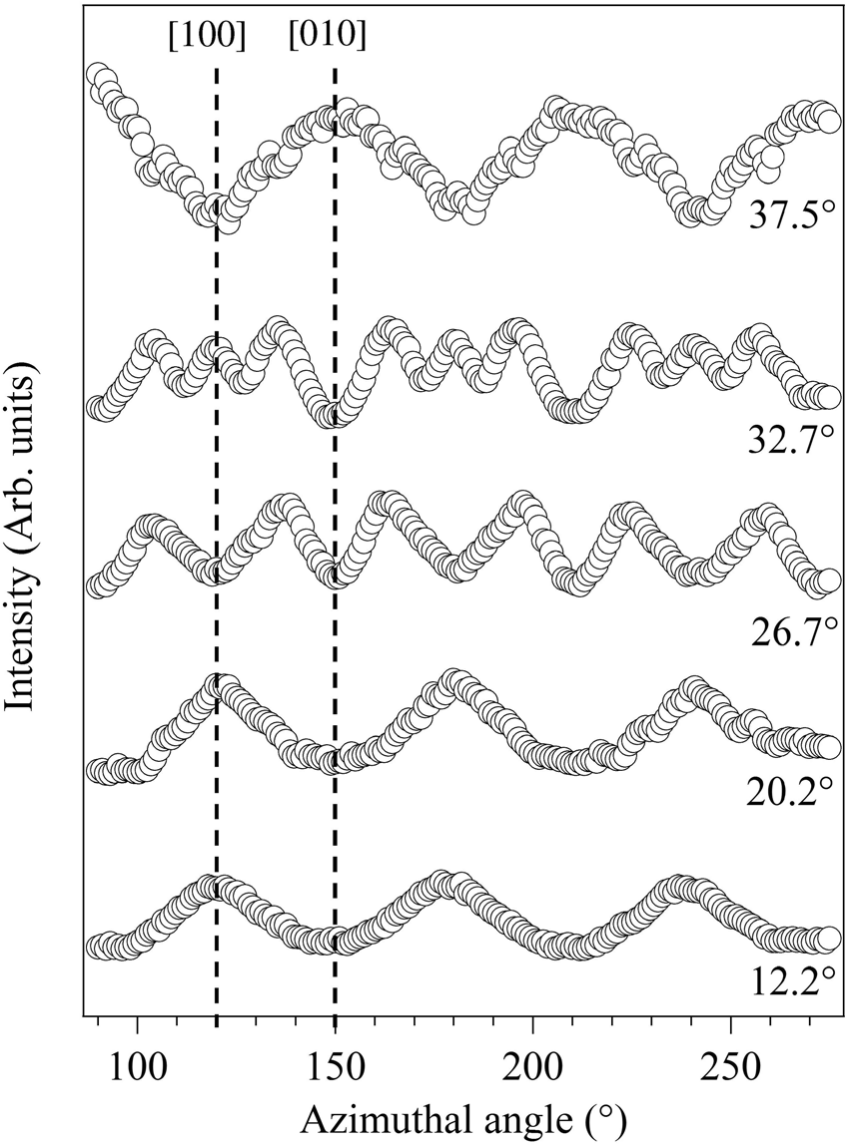
Experimental modulations of the graphene C 1s spectral peak as a function of azimuth at different polar emission angles with a photoelectron energy of 326.1 eV.

In order to extract more quantitative structural information, azimuthal angle-dependent plots of the lower kinetic energy graphene C 1s intensity modulations at specific polar emission angles (marked as white spots in Fig. 5 and selected where strong modulations are seen) were extracted from these full angular distributions. These azimuthal distributions are shown in Fig. 6. Careful inspection of these plots reveals the 3 m symmetry remarked upon above. For example, the shape of the peaks at azimuthal angles of ~150° and ~210° in the plot at a polar angle of 37.5° are not identical, but rather are mirror images of each other. Indeed, most of these azimuthal plots reveal a mirror plane at an azimuth of ~180°.

**Figure 7:**
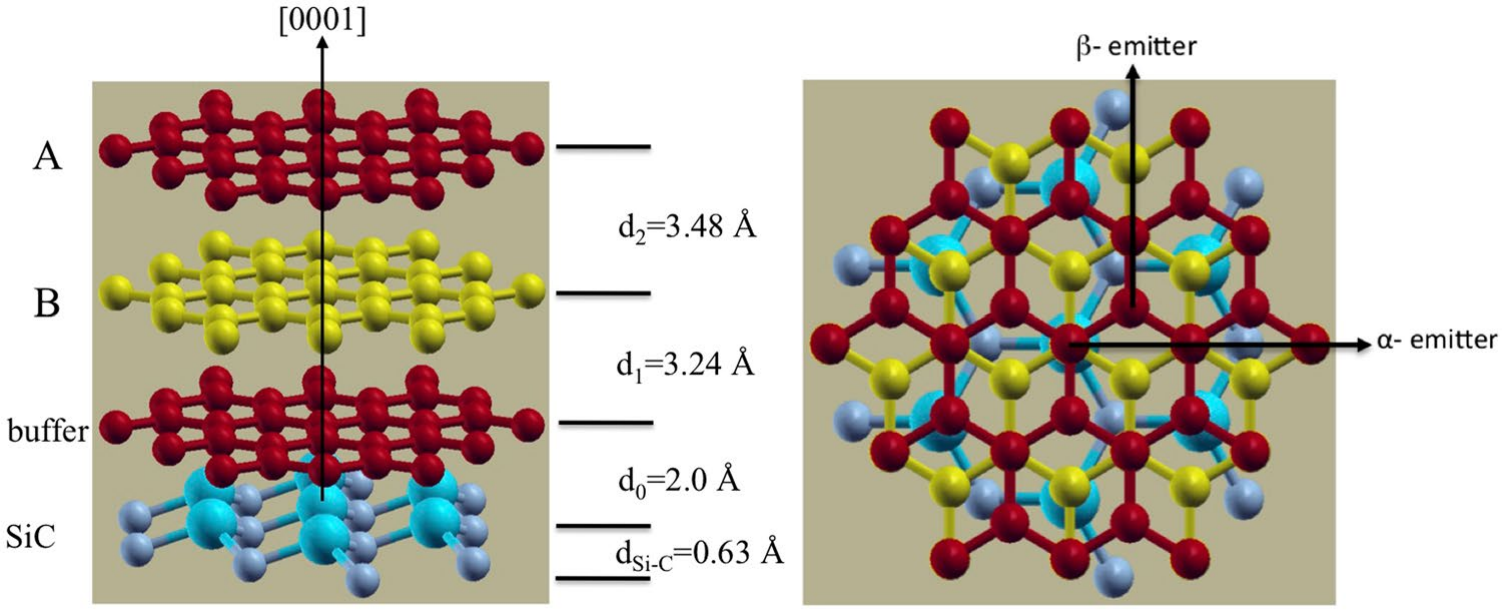
Side view (left) and tow view (right) of the structural model showing the optimised distances.

In order to achieve a more reliable structure determination, based on comparisons with simulated azimuthal distributions for different model structures, the data set was enlarged to cover eight polar emission angles of θ = 12.2°, 20.2°, 26.7°, 32.7°, 37.5°, 46°, 52.5° and 56.8°. Multiple scattering simulations of these photoelectron diffraction azimuthal spectra were based on the structural model depicted in Fig. 7. This comprises two graphene layers stacked in AB sequence above the buffer layer that is rotated by 30° with respect to the SiC substrate. The interlayer distance of 2.0 Å between the SiC substrate and the buffer layer employed in the calculation was based on two previous theoretical studies^22,23^ although other calculations have led to values as large as 2.6 Å^24,25^. The scattering cluster had a radius of 10 Å and contained more than 600 atoms. The two inequivalent α and β type atoms (with and without carbon atom directly below it, respectively) in the hexagonal unit mesh of graphene were used as emitters. Within the graphene bilayer unit mesh there are a total of four C emitters, two each from the first and second graphene layers, that contribute to the graphene C 1s photoemission component. Scattering potentials were calculated within the muffin-tin potential for atoms in a crystal lattice according to the prescription of Mattheis^26^. Atomic mean square displacements of 0.003 Å^2^ were used to account for thermal vibrations by means of Debye-Waller factors. The inner potential was set to V_0_ = −16.5 eV, the value used in the earlier XPD study of this system^21^; because of the relatively high kinetic energies used in our study, small changes in the inner potential have no significant effect on our structural conclusions (see Supplementary Information).

The values of the parameters *d*_1_ (the distance between buffer layer and the first graphene layer) and *d*_2_ (the distance between the first and second graphene layers), as illustrated in Fig. 4, were varied to find the values that gave the best fit of the multiple scattering calculations to the experimental azimuthal plots. The objective test of this best fit was the minimisation of the same reliability factor (*R*) that has been used extensively in scanned-energy mode photoelectron diffraction^27^ defined as

1 $$R=\frac{\sum {({\chi }_{th}-{\chi }_{\exp })}^{2}}{\sum ({{\chi }_{th}}^{2}+{{\chi }_{\exp }}^{2})}$$

where ${\chi }_{th}$ and ${\chi }_{\exp }$ are the theoretical (simulated) and experimental modulation amplitudes and the summations are over all data points in all of the azimuthal plots. Notice that this definition is such that perfect agreement leads to a value of zero while completely uncorrelated values of the simulated and experimental modulations leads to a value of unity.

**Figure 8:**
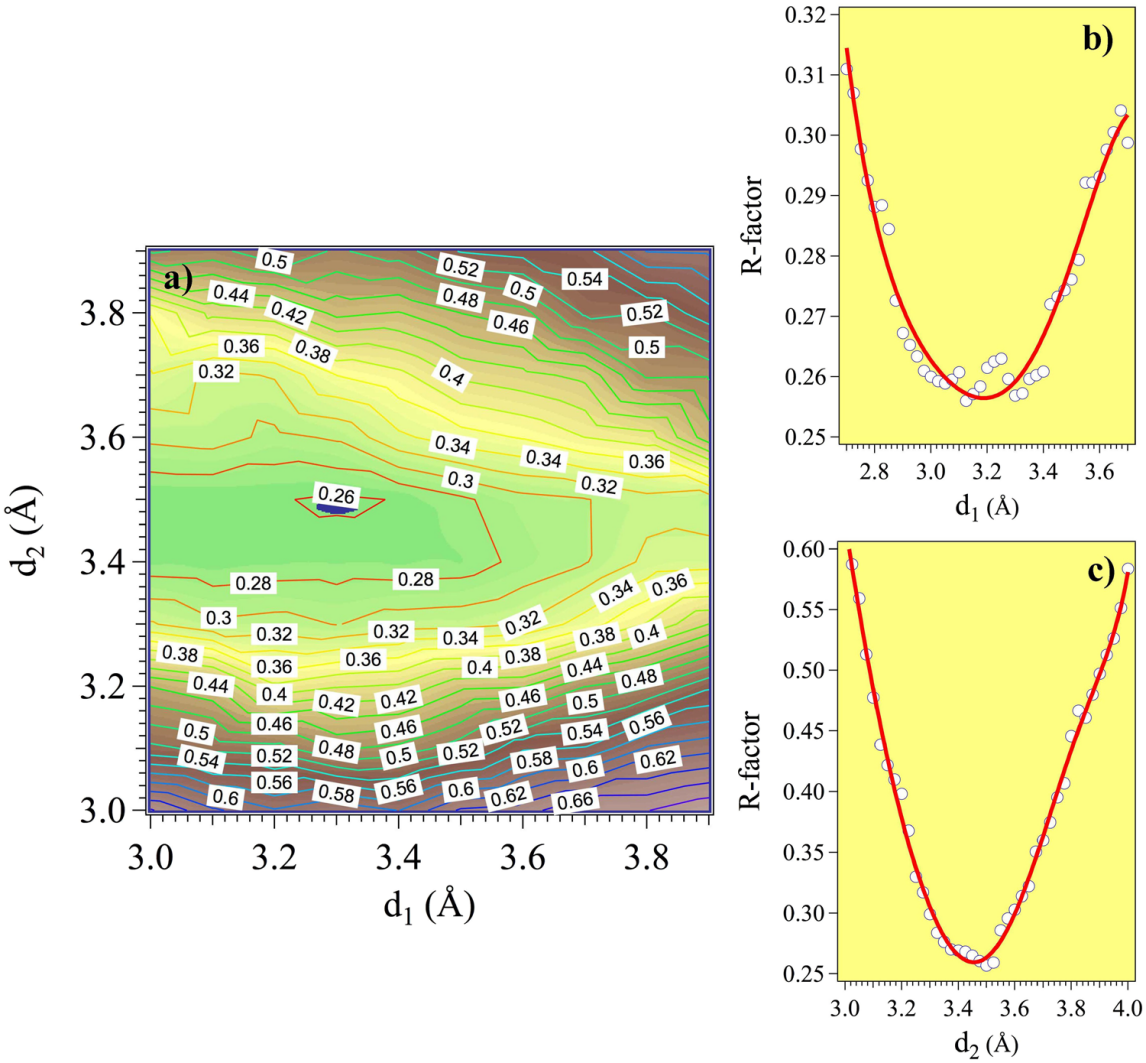
Reliability factor for the AB stacking model when varying the distances between the second and first graphene layers and the buffer layer. (**a**) Shows a two-dimensional false colour map of the *R*- factor, while (**b**) and (**c**) shows the variation with respect to one layer spacing at a fixed value of the other layer spacing as described in the text.

Simulations were performed over a two-dimensional grid scan of *d*_1_ and *d*_2_ in the range 3.00 Å to 3.90 Å, in steps of 0.1 Å, in order to identify the best-fit values. Figure 8(a) shows a false-colour contour plot of the *R*-factor over this grid, and clearly identifies the optimum values of *d*_1_ and *d*_2_ as approximately 3.3 Å and 3.5 Å, respectively. Figure 8(b) shows a one-dimensional grid scan of the variation of *R* as a function of the value of *d*_1_ with the value of *d*_2_ fixed at 3.50 Å, while Fig. 8(c) shows a similar plot of the variation of *R* as a function of the value of *d*_2_ for a fixed value of *d*_1_ of 3.30 Å. Based on these plots we conclude that the best-fit values of the *d*_1_ and *d*_2_ parameters are 3.24 Å and 3.48 Å respectively. In order to estimate the precision of these values we also follow the approach used in scanned-energy mode photoelectron diffraction^27^ of defining a variance in the minimum value of the *R*-factor^28^

2 $$Var({R}_{\min })={R}_{\min }\sqrt{(2/N)}$$

where *N* is the number of independent pieces of structural information within the data being compared. Notice that *N* is not simply the number of data points; the azimuthal plots that form the basis of our experiment/theory comparison clearly show that several data points are required to define a modulation, and it is the number of modulations, or more particularly the possible number of modulations (an absence of a modulation is significant) that define *N*. In scanned-energy mode photoelectron diffraction, the value of *N* can be estimated, as proposed by Pendry^29^ in LEED intensity-energy spectra, by dividing the total energy range by a characteristic peak width related to imaginary part of the inner potential that describes the inelastic scattering. In the present case an equivalent procedure would be to divide the total azimuthal angle range of the data (8 × 180°) by the half-angle of the narrowest observed features, i.e. ~5°. This gives a value of *N* of 288 so as *R*_min_ ≈ 0.26 the variance is ≈0.022 and all values of the structural parameters leading to *R* value less than 0.302 lie within these nominal precision limits. This leads to final values for the two parameters of *d*_1_ = 3.24 ± 0.20 Å and *d*_2_ = 3.48 ± 0.10 Å. The much lower precision in the determination of *d*_1_ is clearly reflected in Fig. 8(a and b). This can be attributed to the fact that the PED from the Gr C 1s emitters is dependent on the location of the buffer layer only through backscattering, and at an electron energy of 326.1 eV the backscattering from the low atomic number C atoms is weak. By contrast, the data are significantly more sensitive to the graphene bilayer interlayer spacing, because this parameter is influenced by forward scattering from the lower-layer emitters as well as backscattering from the outer layer emitters. The optimised model obtained from the simulation is depicted in Fig. 7 showing all the interlayer distances.

Our conclusion that the spacing of the buffer layer and the first graphene layer, *d*_1_, is less than the interlayer spacing in bulk graphite (3.35 Å), and that the interlayer spacing of the graphene bilayer, d_2_, is larger than the value for bulk graphite, is broadly consistent with the results of previous (mainly theoretical) studies. Specifically, our value of d_1_ of 3.24 ± 0.20 Å can be compared with the experimental X-ray reflectivity value of Hass *et al*.^15^ of 2.32 Å, and the theoretical values of Mattausch and Pankratov^24^ (3.3 Å) and of Kim *et al*.^30^ (3.35 Å), while our value of *d*_2_ of 3.48 ± 0.10 Å may be compared with Hass *et al*.’s experimental value of 3.5 Å and the theoretically derived values of values of 3.49 Å^14^, 3.58 Å^13^ and 3.34 Å^11^.

**Figure 9:**
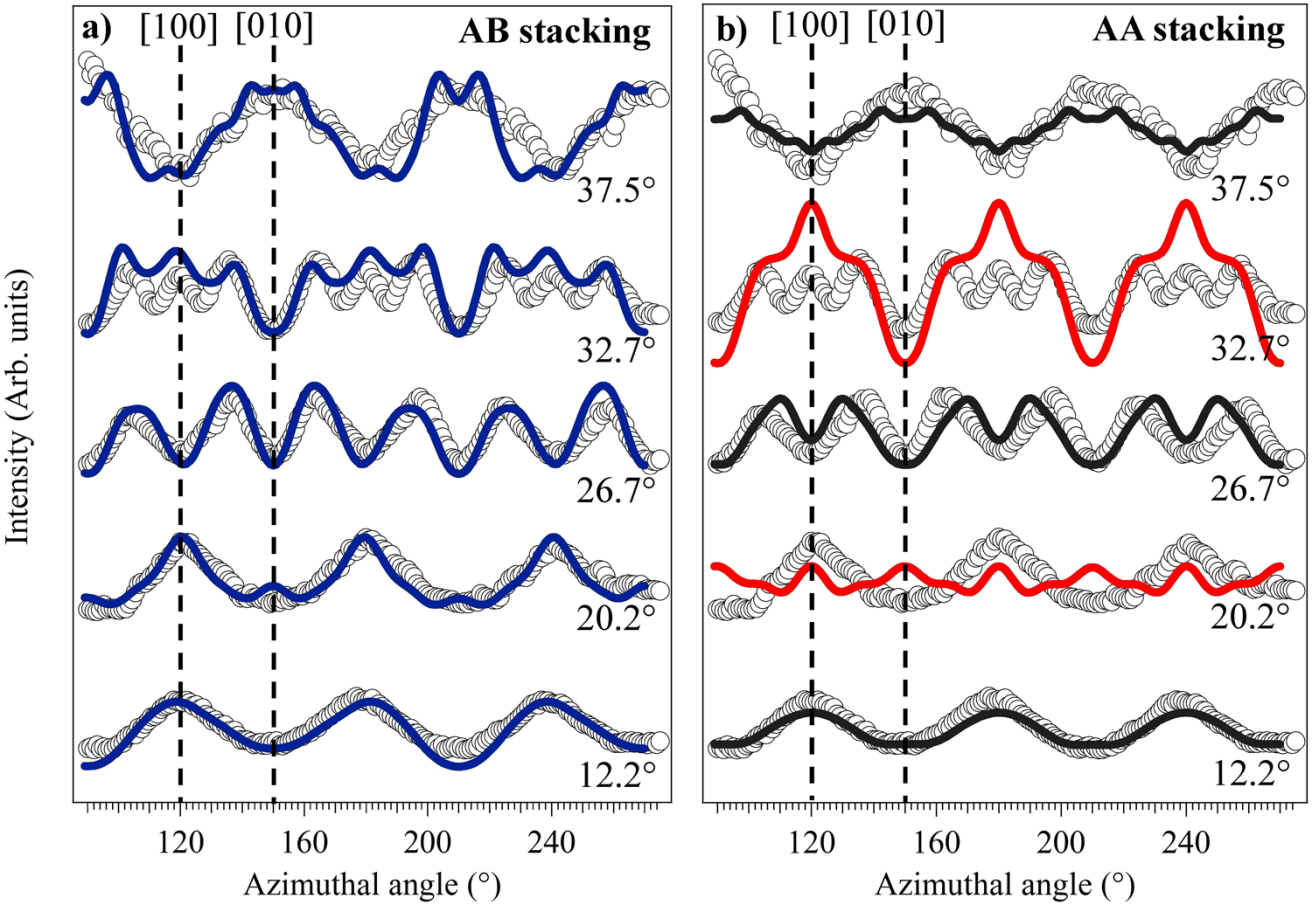
Comparison of experimental (data points) and simulated (continuous lines) intensity modulations of the graphene C 1s photoelectron peak as a function of azimuth at different polar emission angles with a photoelectron energy of 326.1 eV for two different structural models: (**a**) AB stacking and (**b**) AA stacking.

Based on previous theoretical and experimental studies, AB stacking, and not AA stacking, is the equilibrium structure for graphene grown on the Si-terminated SiC(0001) surface. To establish whether our experiments are consistent with this view we have also performed multiple scattering calculations on a model having the AA stacking sequence. Figure 9 shows a comparison of the experimental azimuthal dependence of the graphene C 1s emission at a kinetic energy of 326.1 eV with simulations for both stacking sequences. A very good agreement is achieved by using the optimised *d*_1_ and *d*_2_ values for the AB stacking. By contrast, the simulations for AA stacking clearly show major discrepancies between theory and experiment at polar emission angles of 20.2° and 32.7°; these angles correspond approximately to the first and second nearest neighbour interlayer scattering directions, respectively, for AB stacking (c.f. Fig. 4 in which the smaller value of *d*_2_ of bulk graphite puts these angles at 23° and 36°).

**Figure 10:**
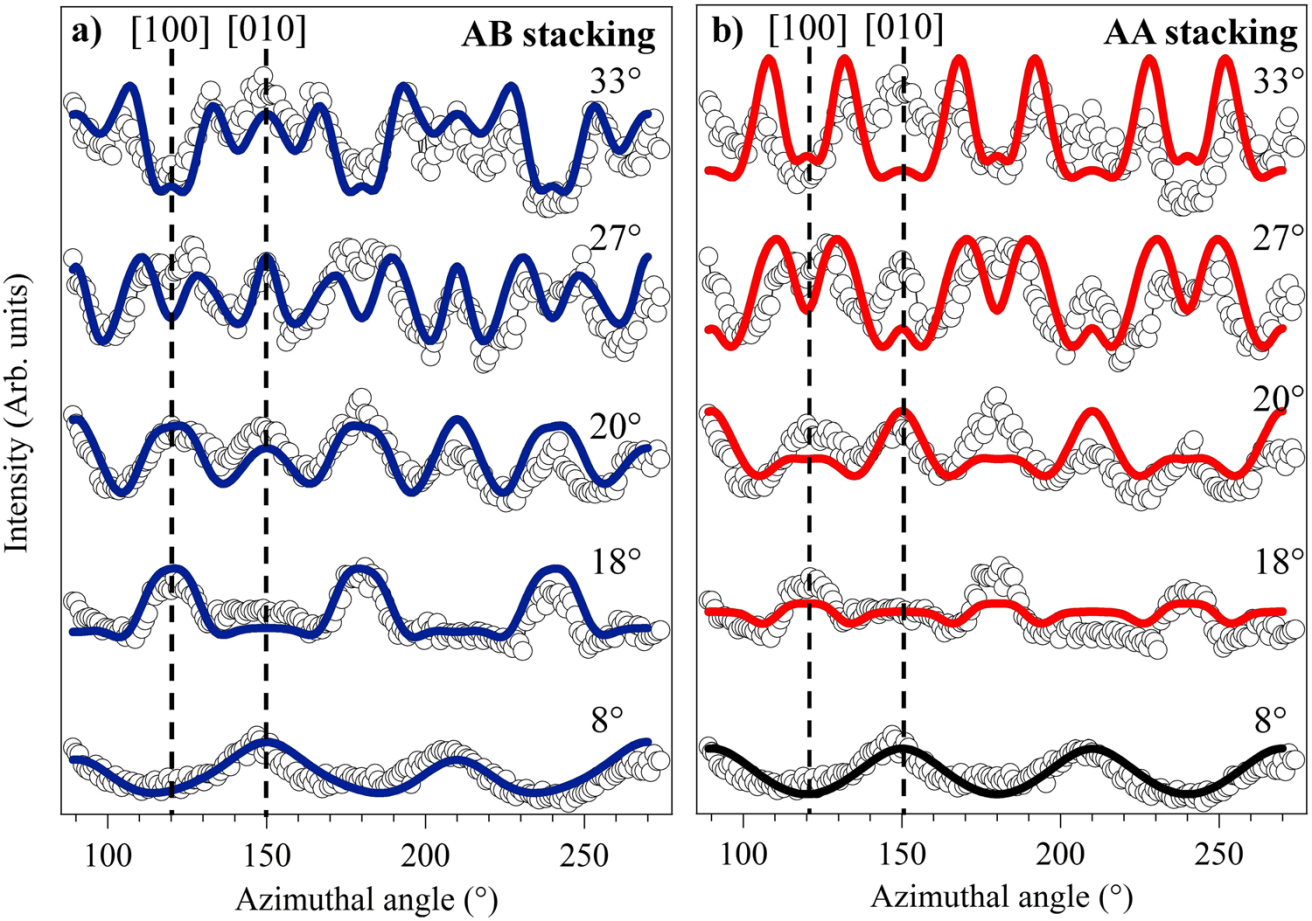
Comparison of experimental (data points) and simulated (continuous lines) intensity modulations of the graphene C 1s photoelectron peak as a function of azimuth at different polar emission angles with a photoelectron energy of 638.6 eV for two different structural models: (**a**) AB stacking and (**b**) AA stacking.

This clear difference in visual comparison is also found in the associated *R*-factor values of 0.26 for AB stacking and 0.45 for AA stacking. Figure 10 shows a similar comparison between experiment and theory for AB and AA stacking models, for a set of graphene C 1s azimuthal scans at the higher photoelectron energy of 638.6 eV. For this data set, too, the AB stacking model yields simulations in very good agreement with experiment, whereas for the simulations based on AA stacking there is particularly poor agreement for the data recorded at 20° and 33° polar emission angles, similarly corresponding approximately to the first and second nearest neighbour forward scattering directions. Clearly these data further reinforce the existing wisdom that bilayer graphene on the Si-face of SiC(0001) has the AB stacking.

## Methods

The photoemission and photoelectron diffraction experiments were carried out at the ANTARES beamline of Synchrotron SOLEIL in Saint-Aubin, France. Photoelectron spectra were obtained using a Scienta R4000 concentric hemispherical analyser equipped with a 2-D detector to allow parallel measurement of a 25° range of collection angles. The sample was placed in the analysis chamber on a sample manipulator with five degrees of freedom, allowing precise orthogonal translation in *x*, *y* and *z* as well as rotation both in polar (θ) and azimuthal (φ) angles. The geometry of the experiments was as follows. The surface normal, the incident beam direction and the electron emission detection direction define a common plane. The angle between the incident beam and the electron energy analyzer is 45°. Measurements of the complete photoelectron diffraction angular distributions were performed by rotating the azimuth of the sample within the 180° range in steps of 1° at specific polar angles. In order to cover a total polar angle range of around 60° this procedure was repeated at polar angles of 19° and 43°. All measurements were recorded with the sample cooled by liquid nitrogen to 120 K. The photoemission and photoelectron diffraction experiments were obtained using an incident photon beam spot size of 140 μm.

The bilayer graphene sample was prepared by the standard graphitisation procedure described in more detail elsewhere^31^. After an initial degassing at 900 °C and surface flattening at 1050 °C, the graphitisation was achieved by holding the SiC substrate at 1220 °C for six minutes, leading to high quality bilayer graphene on the Si-terminated SiC(0001) surface.

Multiple scattering simulations of the measured photoelectron diffraction data were performed using a computer program, based on the magnetic quantum-number expansion formalism, developed by Fritzsche^32^. The computational scheme takes into account the finite energy and angular resolution of the electron energy analyser. In the present calculations single and double scattering events were included. This program is embedded in software that searches for the best agreement between experiment and theory in a multi-dimensional parameter space using an adapted Marquardt algorithm.

## Conclusions

In summary, we have exploited the combination of photoelectron diffraction angular distributions and multiple scattering simulations to show that bilayer graphene grown on the Si-terminated SiC(0001) face has an AB stacking and has an interlayer distance between the buffer layer and the first graphene layer of 3.24 ± 0.20 Å while the interlayer spacing of the bilayer graphene is found to be 3.48 ± 0.10 Å. The smaller spacing between the buffer layer and the first graphene layer than in bulk graphite (3.35 Å), and the fact that the interlayer graphene bilayer spacing is larger than that in bulk graphite, are consistent with several previous theoretical studies. Even if the present results are more precise they are still in agreement with the only other experimental investigation, based on X-ray reflectivity.

## Electronic supplementary material

Supplementary Information
