# Supplementary material for: Structural determination of bilayer graphene on SiC(0001) using synchrotron radiation photoelectron diffraction
Source: Sci Rep. 2018 Jul 5;8:10190. doi: 10.1038/s41598-018-28402-0 (PMC6033894; doi:10.1038/s41598-018-28402-0)
Supplement: Supplementary file 1 — Supplementary Information [file 41598_2018_28402_MOESM1_ESM.pdf]

Supplementary Information: Structural determination of bilayer graphene on SiC(0001) using synchrotron radiation photoelectron diffraction

I. Razado-Colambo,[1, 2#*] J. Avila,[1] D. Vignaud,[3] S. Godey,[3] X. Wallart,[3] D.P. Woodruff,[4] and M.C. Asensio[1*]

[1]Synchrotron SOLEIL, L'Orme des Merisiers, Saint Aubin-BP 48, 91192 Gif sur Yvette Cedex, France

[2]Institute of Mathematical Sciences and Physics, University of the Philippines Los Baños, Laguna 4031, Philippines

[3] Institut d'Electronique, de Microélectronique et de Nanotechnologie (IEMN UMR 8520), Université Lille, CNRS, Centrale Lille, ISEN, Université Valenciennes, F-59000, France

[4]Physics Department, University of Warwick, Coventry CV4 7AL, United Kingdom

*To whom correspondence should be addressed:

* I. Razado-Colambo and M.C. Asensio, corresponding authors, with email addresses ircolambo@up.edu.ph and asensio@synchrotron-soleil.fr
# I. Razado-Colambo permanent address: Institute of Mathematical Sciences and Physics, University of the Philippines Los Baños, Laguna 4031, Philippines

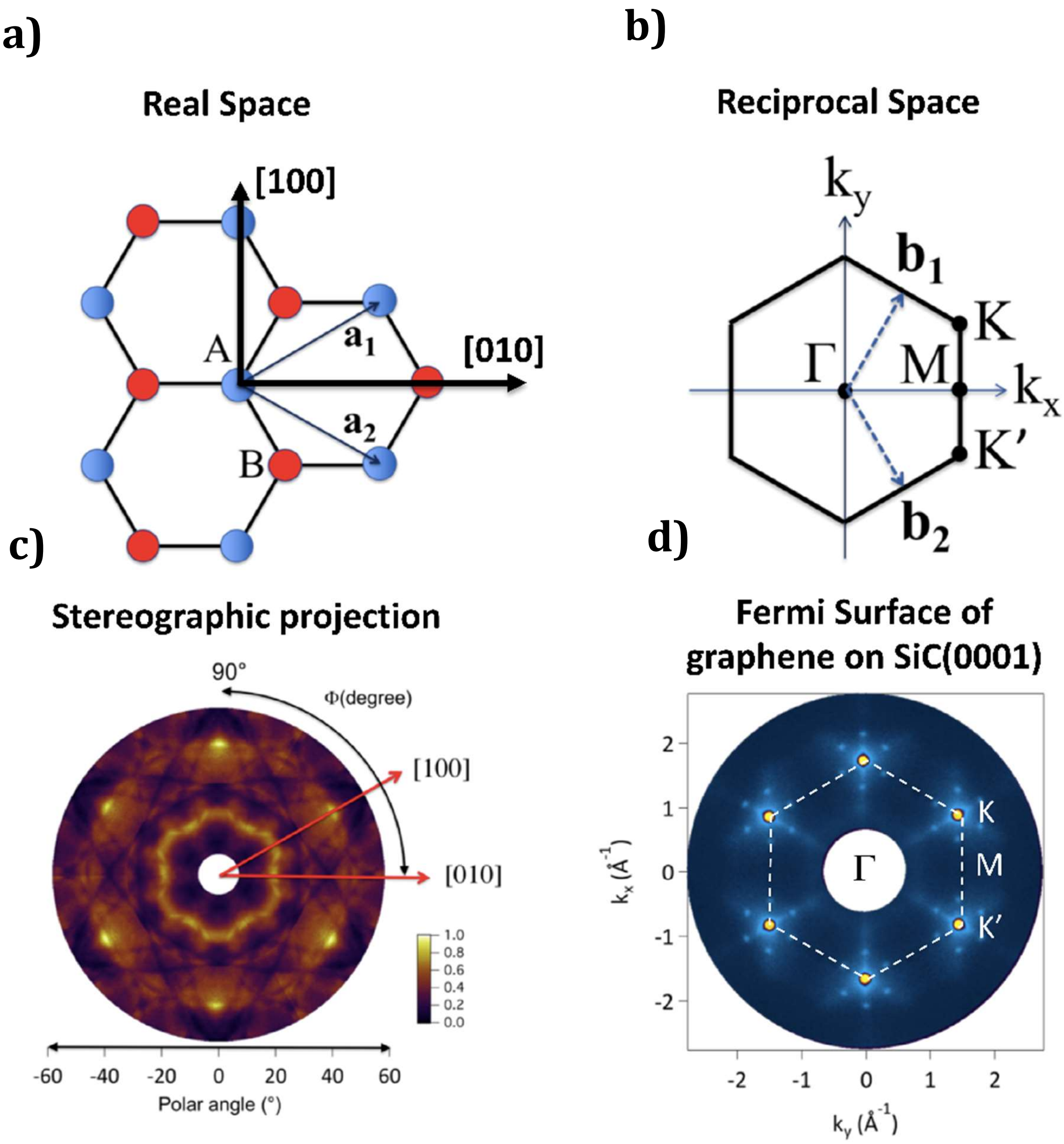

Figure S1. a) Graphene hexagonal unit cell showing the real-space lattice unit vectors and azimuthal directions, b) graphene surface Brillouin zone showing the reciprocal lattice unit vectors and high-symmetry directions, c) stereographic projection of the graphene C 1s photoelectron diffraction pattern from the bilayer graphene on SiC(0001) sample, and d) Fermi surface mapping from exactly the same sample as (c). These images show the unequivocal correspondence of the high-symmetry directions of the real space photoelectron diffraction pattern and the reciprocal space Fermi surface mapping of the same bilayer graphene sample used in this study.

To evaluate the effect of the inner potential on the azimuthal intensity modulations, an investigation of the variation of the *R*-factor as a function of the inner potential wasperformed. Figure S2 shows the dependence of the R-factor on the inner potential in the range from -18.0 eV to -13.0 eV. These calculations were performed using a subset of 5 of the total set of 8 azimuthal plots (a selection that leads to a lower minimum R-factor). The lowest value of the *R*-factor corresponds to V_0= -16.5 eV, but within the range -17.5 eV to -16.0 (the range covered in other publications [i, ii]), the *R*-factor varies by less than 1%. The resulting effect on the calculated photoelectron diffraction curves is therefore minimal, and any change in the resulting structural parameter values is much smaller than our estimated precision.

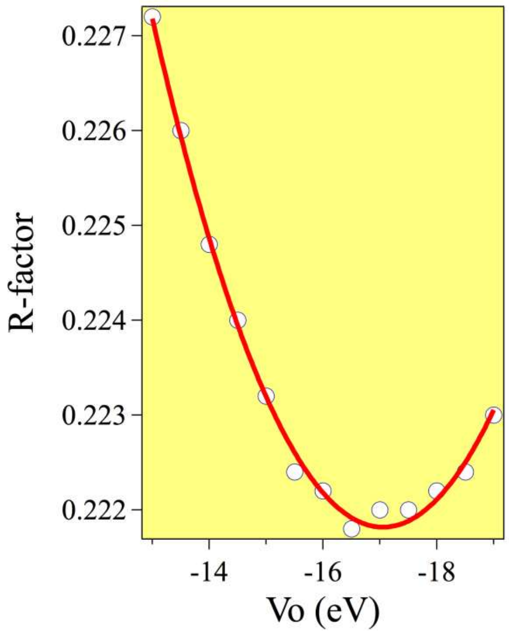

Figure S2. Variation of the R-factor as a function of the inner potential (V_0).

---

i A. Grüneis, C. Attaccalite, T. Pichler, V. Zabolotnyy, H. Shiozawa, S. L. Molodtsov, D. Inosov, A. Koitzsch, M. Knupfer, J. Schiessling, R. Follath, R. Weber, P. Rudolf, L. Wirtz, and A. Rubio, Electron-Electron Correlation in Graphite: A Combined Angle-Resolved Photoemission and First-Principles Study. *Phys. Rev. Lett.* **100**, 037601 (2008).

ii F. Matsui, H. Nishikawa, H. Daimon, M. Muntwiler, M. Takizawa, H. Namba, and T. Greber, The 4πkz periodicity in photoemission from graphite. *Phys. Rev. B* **97**, 045430 (2018).
